# Mutants at the 2-Fold Interface of Adeno-associated Virus Type 2 (AAV2) Structural Proteins Suggest a Role in Viral Transcription for AAV Capsids

**DOI:** 10.1128/JVI.00493-16

**Published:** 2016-07-27

**Authors:** Fikret Aydemir, Maxim Salganik, Justyna Resztak, Jasbir Singh, Antonette Bennett, Mavis Agbandje-McKenna, Nicholas Muzyczka

**Affiliations:** a Department of Molecular Genetics and Microbiology, College of Medicine, University of Florida, Gainesville, Florida, USA; b Department of Molecular Biology and Biochemistry, College of Medicine, University of Florida, Gainesville, Florida, USA; c Powell Gene Therapy Center, College of Medicine, University of Florida, Gainesville, Florida, USA; d Center for Structural Biology, College of Medicine, University of Florida, Gainesville, Florida, USA; e University of Florida Genetics Institute, College of Medicine, University of Florida, Gainesville, Florida, USA; International Centre for Genetic Engineering and Biotechnology

## Abstract

We previously reported that an amino acid substitution, Y704A, near the 2-fold interface of adeno-associated virus (AAV) was defective for transcription of the packaged genome (M. Salganik, F. Aydemir, H. J. Nam, R. McKenna, M. Agbandje-McKenna, and N. Muzyczka, J Virol 88:1071–1079, 2013, doi: http://dx.doi.org/10.1128/JVI.02093-13). In this report, we have characterized the defect in 6 additional capsid mutants located in a region ∼30 Å in diameter on the surface of the AAV type 2 (AAV2) capsid near the 2-fold interface. These mutants, which are highly conserved among primate serotypes, displayed a severe defect (3 to 6 logs) in infectivity. All of the mutants accumulated significant levels of uncoated DNA in the nucleus, but none of the mutants were able to accumulate significant amounts of genomic mRNA postinfection. In addition, wild-type (wt) capsids that were bound to the conformational antibody A20, which is known to bind the capsid surface in the region of the mutants, were also defective for transcription. In all cases, the mutant virus particles, as well as the antibody-bound wild-type capsids, were able to enter the cell, travel to the nucleus, uncoat, and synthesize a second strand but were unable to transcribe their genomes. Taken together, the phenotype of these mutants provides compelling evidence that the AAV capsid plays a role in the transcription of its genome, and the mutants map this functional region on the surface of the capsid near the 2-fold interface. This appears to be the first example of a viral structural protein that is also involved in the transcription of the viral genome that it delivers to the nucleus.

**IMPORTANCE** Many viruses package enzymes within their capsids that assist in expressing their genomes postinfection, e.g., retroviruses. A number of nonenveloped viruses, including AAV, carry proteases that are needed for capsid maturation or for capsid modification during infection. We describe here what appears to be the first example of a nonenveloped viral capsid that appears to have a role in promoting transcription. A total of six mutants at the AAV capsid 2-fold interface were shown to have a severe defect in expressing their genomes, and the defect was at the level of mRNA accumulation. This suggests that AAV capsids have a novel role in promoting the transcription of the genomes that they have packaged. Since wt virions could not complement the mutant viruses, and the mutant viruses did not effectively inhibit wt gene expression, our results suggest that the capsid exerts its effect on transcription in *cis*.

## INTRODUCTION

Adeno-associated virus (AAV) has one of the smallest known virus particles, with a diameter of ∼26 nm and T=1 symmetry (1). AAV is currently in widespread use as a gene delivery vehicle for the therapy of a variety of human diseases and as a research tool for developing new animal models of disease (2). The capsid is composed of three proteins, VP1, VP2, and VP3, which are present at a ratio of about 1:1:10. All three capsid proteins share a coding sequence, with the larger capsid proteins, VP1 and VP2, having additional N-terminal sequences. The virus enters cells through several kinds of receptors into an early endosomal compartment. Acidification of the virus in an endosomal compartment is essential for the extrusion of the N terminus of the largest capsid protein, VP1, which contains nuclear localization signals as well as a phospholipase activity that is thought to be involved in endosomal lysis and escape of the capsid into the cytoplasm. The capsid then travels to the nucleus via either the Golgi membrane or microtubules and is believed to enter the nucleus as an intact capsid. Once in the nucleus, the single-stranded viral DNA is uncoated, and a second strand is synthesized. The duplex DNA is then transcribed, and its genes are expressed.

**FIG 1:**
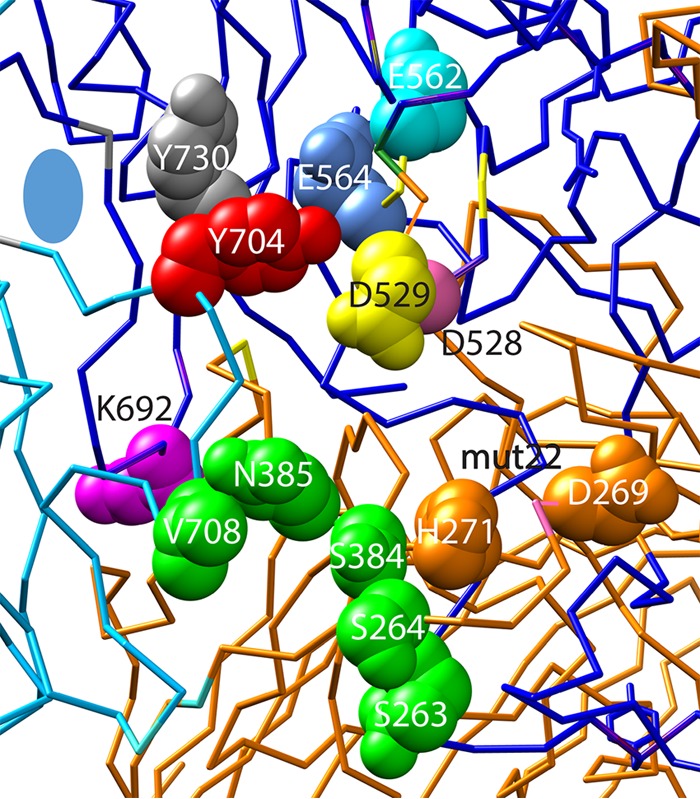
Positions of the mutations in the 30-Å-diameter dead zone. The relative positions of the E562 (cyan), E564 (blue), D528 (yellow), D529 (pale red), and K692 (purple) mutants and the double mutant mut22 (H271 D269) (orange), which were characterized in this study, are shown on the atomic structure of AAV2. The wt amino acids that were mutagenized are shown as space-filling molecules on one side of the 2-fold interface (blue oval) on the peptide chain backbone of this region of the capsid. The dark blue and cyan chains form the 2-fold interface. The orange peptide chains form a 3-fold interface with the reference blue chain. Also shown are the residues (green) that were previously shown to be involved in binding to the A20 antibody (3). In addition, Y704 (red) was previously shown to eliminate transcription when converted to an alanine residue (6), and Y730 (gray) was previously shown to increase transduction when converted to a phenylalanine residue (7).

In previous work, we and others (3, 4) have found a number of mutants that are capable of assembling intact capsids but have a profound defect in infectivity (3 to >5 logs). These mutants cluster in a 30-Å-diameter region adjacent to the 2-fold interface on the capsid surface (Fig. 1) that has been called the “dead zone” (3). This region has been shown by X-ray structure analysis to be one of two regions that change their configuration when the virus is subjected to acidic pHs (pH <5) (5), suggesting that this region may be responsible for the structural change that results in the extrusion of the VP1 N terminus. In addition, recent work has identified another potential activity in the 2-fold region. A nearby Y704A mutation (Fig. 1, red) produces a capsid that appears to be capable of accumulating in the nucleus, uncoating, and undergoing second-strand synthesis but is defective in transcribing its DNA (6). In contrast, a capsid with a Y704F substitution shows a modest increase in gene expression, and a nearby Y730F substitution shows a significant increase in gene expression (7). Our hypothesis was that all of the mutants at the 2-fold interface that had previously been identified as being severely defective for infectivity would share the Y704A defect for transcription.

In this study, we characterize the defects in six additional mutants that were previously identified in the 2-fold region. We find that all of the mutants in this region are capable of cell entry, nuclear accumulation, uncoating, and second-strand synthesis. Instead, like Y704A, these mutants are defective in the transcription of the genome after second-strand synthesis. This also appears to be the mechanism of neutralization by the structure-specific monoclonal antibody A20. Taken together, data from our studies suggest that the 2-fold region of the AAV capsid has a role in transcription of the viral genome.

## MATERIALS AND METHODS

### Cell culture.

HEK293T and HeLa cells were maintained at 5% CO_2_ and 37°C in Dulbecco modified essential medium and F-12K medium (ATCC, Manassas, VA), respectively, supplemented with 10% fetal bovine serum, 100 U/ml penicillin, and 100 mg/ml streptomycin.

### Site-directed mutagenesis.

Overlapping forward and reverse primers with the mutated sequences were used to amplify the pIM45 plasmid by using high-fidelity DNA polymerase (New England BioLabs, Ipswich, MA). Amplification products were digested with DpnI, transfected into electrocompetent Escherichia coli NEB 5-alpha cells, and selected on plates containing ampicillin. All mutants were sequenced to confirm the mutation.

### Virus production.

Mutant virus stocks were prepared as previously described (6). Lipofectamine or polyethyleneimine transfection methods were used for small-scale and large-scale preparations, respectively. Either wild-type (wt) or mutant pIM45, pXX6, and either pTR-UF11 (single-stranded enhanced green fluorescent protein [eGFP] genome), pds-eGFP (double-stranded eGFP genome), or pTR Luc-cherry (expressing mCherry plus luciferase [Luc] genomes) were transfected into HEK293T cells, and viral lysates were collected at 68 h posttransfection. Viral lysates were treated with Benzonase, clarified by centrifugation, and purified by iodixanol gradient centrifugation. The titers of the viral stocks were determined by using quantitative PCR (qPCR) with SYBR select master mix (Thermo Scientific, Grand Island, NY), with forward primer TGA TGC CAC ATA CGG AAA GC and reverse primer AAA AGC ACT GCA CGC CAT AG. Titers of self-complementary-genome-carrying viruses were determined with forward primer GCA TCG ACT TCA AGG AGG AC and reverse primer ATG CCG TTC TTC TGC TTG TC.

### Infectivity assay.

HEK293T cells were seeded at 1 × 10^4^ cells per well into 96-well plates 12 h prior to infection. Cells were infected in triplicate at a multiplicity of infection (MOI) of 10 to 10,000 DNA-containing particles per cell and coinfected with adenovirus type 5 (Ad5) at an MOI of 10. Both wt and mutant capsids contained the same GFP cassette, in either a single-stranded DNA (ssDNA) form or a self-complementary form. Adenovirus coinfection was used to rapidly promote second-strand synthesis and gene expression and to simulate productive AAV infection conditions. In the presence of Ad coinfection, there was no significant difference in gene expression at 24 h between single-stranded and self-complementary genomes. The wells were photographed by using an Axiovert 100 fluorescence microscope (Zeiss, Peabody, MA), and the number of green cells and the total number of cells were counted from these images by using ImageJ software (8). The particle-to-infectivity ratio (the number of input genomes divided by the number of green cells) was calculated to determine the minimum number of genomes required to produce one transduced cell that expressed GFP.

### Subcellular fractionation.

HeLa cells were seeded onto 6-well plates 12 h prior to infection. Cells were infected with wt or mutant AAV at an MOI of 10,000 and with Ad5 at an MOI of 10 in 1 ml of complete medium at 4°C for 30 min. The cells were then shifted to 37°C. At the appropriate time, cells were collected by trypsinization, pelleted in a refrigerated centrifuge at 250 relative centrifugal force (RCF), and washed three times with ice-cold 1× phosphate-buffered saline (PBS). Subcellular fractionation was performed by using NE-PER nuclear and cytoplasmic extraction reagents (Thermo Scientific, Grand Island, NY) according to the manufacturer's instructions. The fractionation process resulted in cytoplasmic and nuclear fractions as well as a postnuclear pellet that contained the nuclear membrane and cellular DNA. Each fraction was adjusted to 200 μl (postnuclear pellet suspended in 200 μl) of water, and the nuclear and postnuclear fractions were combined and referred to as the nuclear fraction.

### qPCR.

Viral DNA purification was performed by using a QIAquick PCR purification kit (Qiagen, Valencia, CA) according to the manufacturer's recommendations, with slight modifications. Iodixanol (Sigma-Aldrich Corp., St. Louis, MO) fractions from virus purification or subcellular fractions from HeLa cell lysates were incubated with 5 volumes of PB buffer containing 150 μM sodium acetate (NaOAc) at room temperature for 10 min before the procedure was started to provide complete denaturation of the capsid proteins. This modification kept the solutions acidic to provide maximum DNA binding for subsequent steps. Viral DNA that was released from virions was captured with silica-based columns at an acidic pH of 5.3. Following 5 min of ethanol incubation and washing, captured DNAs were released from the silica membrane by using 100 μl elution buffer, which had 100 μM EDTA added for a higher pH of 8.0. Eluted DNA was used directly to quantify copy numbers of viral genomes in the solutions by using qPCR with the primers listed above.

### DNase protection assay.

For assessment of the relative portion of uncoated virions in the nucleus, cells were prepared, infected, and fractionated as described above. Once nuclear fractions (soluble nuclear fraction and postnuclear pellet) were obtained by using the NE-PER kit (Thermo Scientific, Grand Island, NY), fractions were adjusted to 200 μl with Benzonase buffer (10 mM Tris [pH 7.5], 5 mM MgCl_2_) and then split in half. One half was processed as detailed above for subcellular fractionation to obtain the total amount of DNA, while the other half was subjected to digestion with 250 U of Benzonase (Sigma-Aldrich, St. Louis, MO) for 1 h at 37°C. Viral DNA was then purified as described above. Both the total and Benzonase-treated samples were subjected to real-time PCR (RT-PCR) with GFP primers as described above, and the relative fraction of DNase-resistant genomes as well as the absolute mean number of DNase-sensitive genomes per cell were calculated.

### RT-PCR assay.

HeLa cells were seeded at 1 × 10^5^ cell per well in 6-well plates 24 h prior to infection. Cell were infected with 1 × 10^4^ viral genomes (vg)/cell of either wild-type or mutant AAV packaged with pRT-UF11 and coinfected with Ad5 (MOI = 10), as described above. At 24 h postinfection (p.i.), cells were collected, and the total cellular fraction was purified by using TRIzol RNA reagent (Life Technologies, Grand Island, NY). RNA integrity was assessed by using an Agilent 2100 bioanalyzer, and all samples were found to have an RNA integrity number (RIN) of 9 or higher, after using a ProtoScript II first-strand cDNA synthesis kit (New England BioLabs, Ipswich, MA). The number of AAV transcripts was determined by real-time PCR (MyIQ; Bio-Rad, Hercules, CA), using primers against the GFP portion of pTR-UF11 (forward primer TGA TGC CAC ATA CGG AAA GC and reverse primer AAA AGC ACT GCA CGC CAT AG). pTR-UF11 plasmid DNA linearized with ScaI was used as a standard for determining copy numbers. Calculation of the number of GFP mRNA copies per cell was done by using the StepOnePlus real-time PCR system (Thermo Scientific, NY) and Excel.

### Sequence and atomic structure comparisons.

Sequence alignments of all previously reported AAV serotypes was performed with FastA using sequences in GenBank. Structural alignments were performed by using PyMOL and previously reported PDB data for AAV type 1 (AAV1), AAV2, AAV4, and AAV5.

### Statistical analysis.

**FIG 2:**
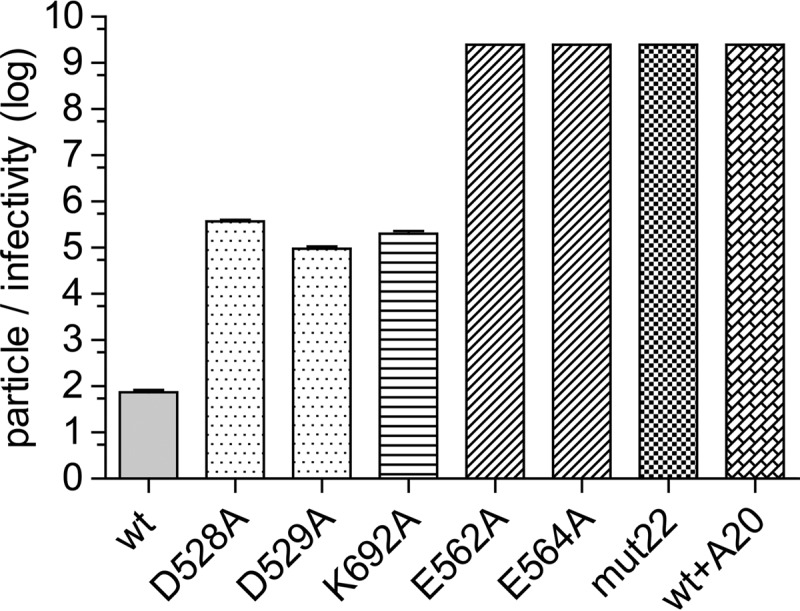
Particle-to-infectivity ratios of mutants carrying a single-stranded GFP expression cassette. Shown are log particle-to-infectivity ratios of the six mutants in this study compared to the wt and the wt complexed with the A20 antibody. The particle-to-infectivity ratio is the ratio of the number of DNase-resistant particles per milliliter (determined by RT-PCR) divided by the number of transduced green cells per milliliter of virus in the presence of a coinfecting adenovirus. Each bar represents the mean of data from three experiments; error bars indicate standard errors. Mutants with no error bars showed no detectable infectivity, and therefore, the particle-to-infectivity ratio is >2.5 × 10^9^. One-way ANOVA was used to determine statistical significance (*F*[7,16] = 68; *P* < 0.0001; *n* = 3). Tukey's *post hoc* results indicated that all comparisons with the wt had *P* values of <0.001, except for comparisons of the wt versus the D529A mutant, where the *P* value was <0.01.

**FIG 3:**
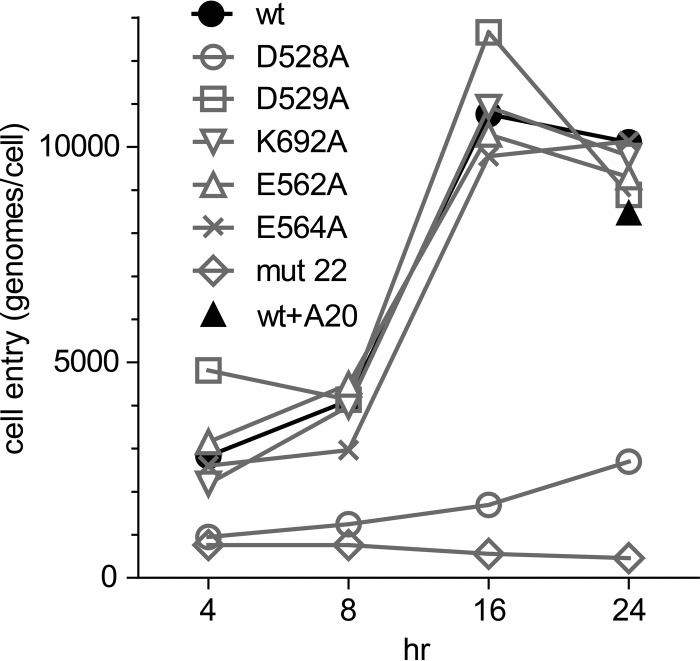
Time course of entry of wt and mutant cells. HeLa cells were infected with wt and mutant viruses at an MOI of 10,000 as described in Materials and Methods. At the indicated time points, intracellular viral DNA was collected, and the number of genomes that had entered per cell was measured by RT-PCR. Most of the mutants (gray lines) entered the cells as well as the wt. The wild-type virus bound to the A20 antibody (1:17 ratio) accumulated in cells at about the same rate as that of the wt. Two mutants (D528A and mut22) were significantly defective for cell entry. Data for each time point for each mutant represent the means of results from three separate experiments. Error bars for each time point were omitted for clarity.

**FIG 4:**
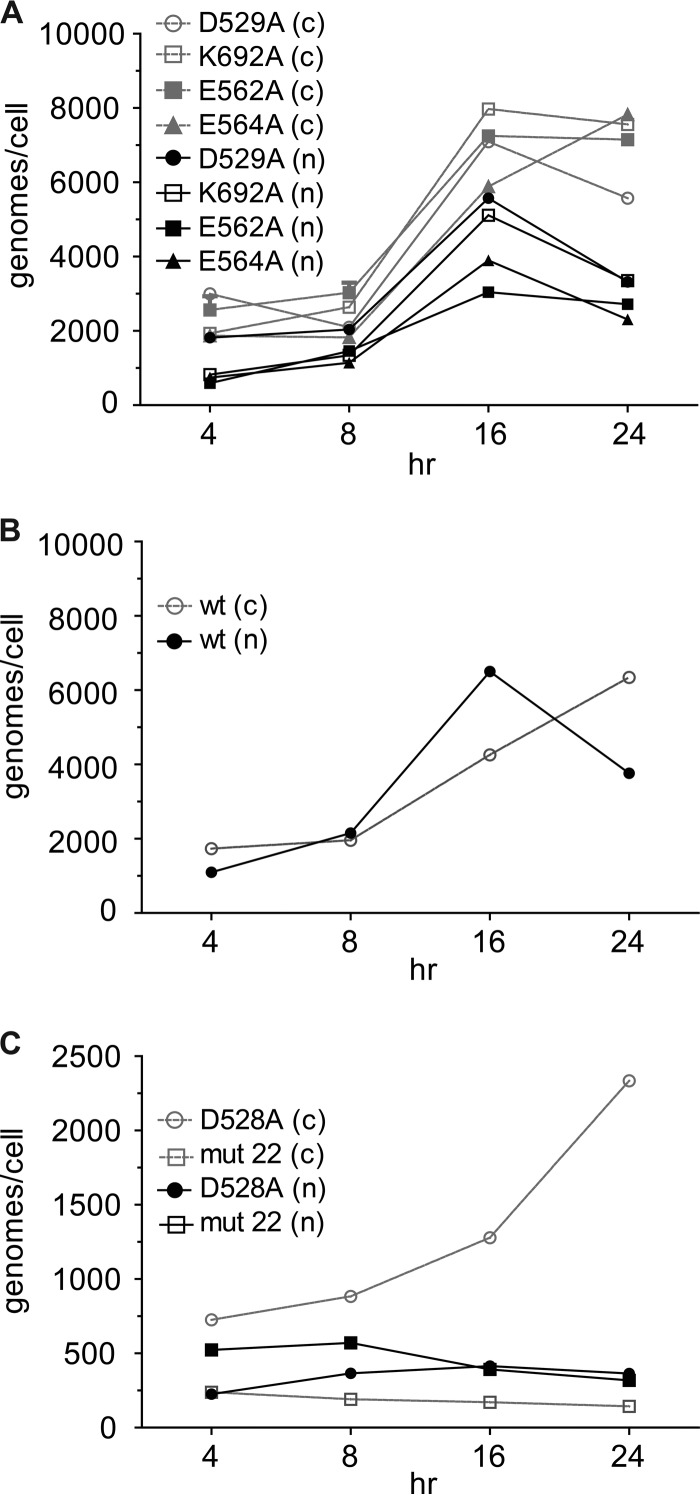
Distribution of virus between the nucleus (n) and cytoplasm (c). Mutant viruses that showed little or no defect for cell entry distributed themselves between the nucleus and cytoplasm approximately the same as the wt virus (compare panels A and B) as a function of time. In contrast, the D528A mutant showed a much higher level of retention of virus in the cytoplasm over time, while mut22 showed a much higher relative level of accumulation of virus in the nucleus than the wt (compare panels B and C). Data for each time point are the means of results from three separate experiments; error bars were omitted for clarity.

With the exception of the complementation experiment shown in Fig. 9, all experiments for all mutants were done at least three independent times, and PCR determination for each independent mutant experiment was also done in triplicate. The mean plus standard error for each mutant were determined and plotted. Values for some mutants, notably those shown in Fig. 2 and Fig. 7, were below the level of detection, and error bars were omitted in these cases. These mutants are noted in the figure legends. Error bars were also omitted in Fig. 3 and 4 for clarity. Statistical analysis was done by using Graph Pad Prism 5. Where indicated in the figure legends, statistically significant differences between mutants were determined by using one-way analysis of variance (ANOVA) with Tukey's *post hoc* analysis for comparison of individual mutants to the wt.

## RESULTS

Both Wu et al. (4), who made a series of charged cluster mutants in the AAV2 capsid, and Lochrie et al. (3), who targeted surface residues, found a set of mutations that assembled capsids but had a severe defect in infectivity. These mutants clustered at the capsid 2-fold interface. The mutants described by Wu et al. (4) included mut22 (D269A,H271A), mut37 (^527^KDDEEK^532^ to ^527^AAAAAA^532^), mut40 (^561^DEEE^564^ to ^561^AAAA^564^), and mut47 (^689^ENSKR^693^ to ^689^ASSAA^693^). Subsequent work by Lochrie et al. (3) and Salganik et al. (6) identified the individual residues within mut37 and mut40, respectively, that were likely to account for the phenotype seen by Wu et al. as D528A,D529A and E562A,E564A. In addition, Lochrie et al. (3) confirmed that the single mutations H271A and D269A, which together constituted mut22 (Fig. 1), were 2 and 2 logs defective, respectively. Finally, recent unpublished work from our laboratory also identified K692A as the likely cause of the defect in mut47. This mutant had previously been characterized as being defective for capsid assembly but was subsequently found instead to be defective for binding the A20 antibody, which was the assay used for capsid assembly in the original study by Wu et al. We therefore focused our analysis on the six mutants D528A, D529A, K692A, E562A, E564A, and mut22 (Fig. 1). With the exception of mut22, none of the mutant sequences overlapped the amino acid sequence of the assembly-activating protein (AAP), which is contained within the capsid reading frame and is read in an alternate reading frame. The two mutations in mut22 (D269A,H271A) were silent mutations in AAP, producing no amino acid changes. All of the mutants produced virus titers that were within a factor of 2 of wild-type titers. In addition, K692A and mut22 were both near a set of amino acids identified previously by Lochrie et al. (3) as being involved in binding neutralizing antibody A20 (Fig. 1, green residues). A20 was identified as a conformational antibody that interrupted AAV infection after the capsid entered the nucleus (9–11), and we therefore also characterized the defect in virus bound to the A20 antibody. All of the mutants, as well as the A20 binding site, are within a 30-Å-diameter region that Lochrie et al. (3) called the dead zone.

### Mutant-particle-to-infectivity ratios.

As mentioned above, we recently characterized the defect in a potential dead-zone mutant, Y704A, and found that although Y704A accumulated in the nucleus and was uncoated, there was virtually no transcription of the recombinant genome (6). To see if the defect in other dead-zone mutants was similar, we first determined the particle-to-infectivity ratio for each mutant (Fig. 2). The D528A and D529A mutants transduced HeLa cells ∼3 logs less effectively than did wt capsids containing the same GFP cassette. This was in contrast to the previously reported 2-log and 5-log defects, respectively (3). K692A also had a ∼3-log defect for transduction. Previously, a compound mutant that contained the K692A mutation, mut47, had a more severe 5-log defect for transduction (4), suggesting that the mut47 phenotype was due not only to the K692A mutation but also to three other mutations present in mut47. In addition, E562A, E564A, and mut22 all had a 6- to 7-log defect, confirming previously reported determinations (6).

### Cell entry.

To see if any of the mutants had a defect in cell entry, we bound each mutant to HeLa cells at 4°C for 30 min and then raised the temperature to 37°C to initiate cell entry. Each mutant was applied to cells at an MOI of ∼10,000 particles per cell, and cells were coinfected with Ad5 at an MOI of 5. Adenovirus coinfection was used to induce rapid second-strand synthesis of the GFP cassette and to simulate the cellular conditions during productive AAV infections. However, since no *rep* genes were present or expressed, there was no amplification of the input genomes. At various times after the temperature was raised, we washed the cells and determined the total number of intracellular genome copies per cell as a function of time by RT-PCR. Most of the mutants entered the cell at approximately the same rate as that of the wt, with a peak at 16 h, at which point nearly all of the particles had entered the cells (Fig. 3). This was also true of wt particles containing the GFP cassette that had been preincubated with the A20 antibody at an antibody-to-capsid ratio (1,700:1 molar ratio; ∼28 antibody molecules per capsid protein monomer) that had been shown to completely inhibit transduction. In contrast, D528A and mut22 entered the cells poorly (Fig. 3), accumulating in cells at ∼25% and 10% of wt levels, respectively. Thus, the defects in D528A and mut22 were due at least in part to their reduced ability to enter cells.

### Nuclear accumulation.

**FIG 5:**
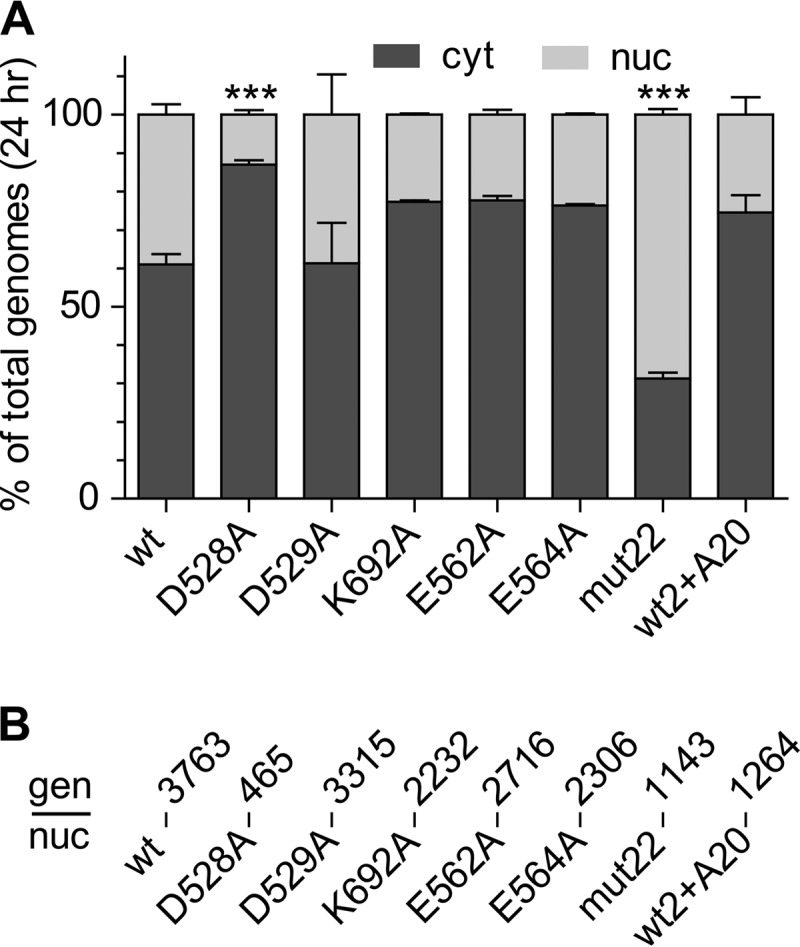
Efficiency of nuclear entry. (A) Percentages of total viral genomes in the cell that entered the nucleus (nuc) by 24 h. Two mutants, D528A and mut22, accumulated significantly lower and higher percentages of genomes, respectively, in the nucleus than the wt, as determined by one-way ANOVA (*F*[7,17] = 17.37; *P* < 0.0001; *n* = 3). Tukey's *post hoc* comparisons to the wt are indicated (***, *P* < 0.001). There was no significant difference between the remaining mutants or the A20-bound virus and the wt. cyt, cytoplasm. (B) The number of viral genomes per nucleus (gen/nuc) is indicated for each mutant or the A20-bound wt virus (top row) at 24 h (means of data from three experiments) regardless of variations in cell entry or nuclear accumulation rates.

To see if the mutant particles that entered the cell were able to accumulate in the nucleus, we fractionated cells to obtain the cytoplasmic and nuclear fractions. The numbers of genome copies in each compartment were then measured by RT-PCR. The mutants segregated into three categories. Most of the mutants entered the nucleus with a time course that was similar to that of the wt (compare Fig. 4A and B), with a peak in the number of genome copies per nucleus at between 16 and 24 h. In contrast, the D528A mutant appeared to be significantly impaired in nuclear accumulation, and mut22 was significantly better at nuclear accumulation than the wt (Fig. 4C and 5A). When the percentage of total intracellular genomes that entered the nucleus was determined at 24 h (Fig. 5A), only D528A and mut22 were significantly different from the wt. Approximately 15% of the intracellular D528A mutant entered the nucleus, while almost 75% of mut22 entered the nucleus. In contrast the wt and the other mutants (including the wt complexed with A20) accumulated 25 to 40% of their intracellular genomes in the nucleus.

We also calculated the number of genomes that entered per nucleus for each mutant. Given that most of the mutants showed no significant defect in entering the cell or subsequently entering the nucleus, it was not surprising that the number of genomes per nucleus for most of the mutants varied by only 2.5-fold, with 1,264 genomes for the wt bound to A20 and 3,763 genomes for the wt (Fig. 5B). Even the two mutants that had a defect for cell entry, D528A and mut22, accumulated a significant number of genomes per nucleus, 365 and 1,143, respectively. Thus, the reduced transduction displayed by the mutants (Fig. 2), including D528A and mut22, could not simply be ascribed to defects in cell or nuclear entry.

### Uncoating in the nucleus.

**FIG 6:**
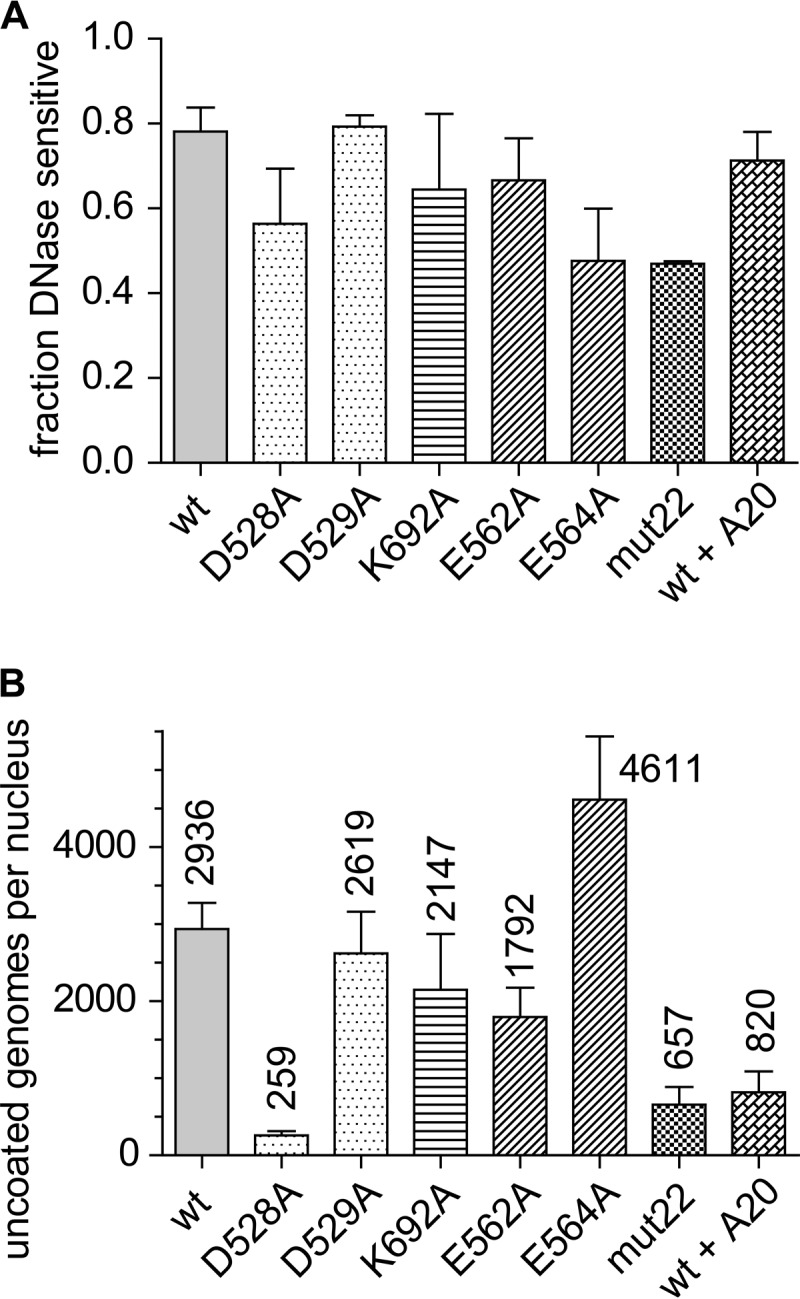
DNA uncoating in the nucleus. (A) Fractions of genomes in the nucleus that were DNase sensitive at 24 h and therefore considered uncoated. One-way ANOVA showed no significant difference in the rates of DNA uncoating once the mutants were in the nucleus. Error bars show standard errors of data from three separate experiments. (B) Numbers of wt or mutant genomes that were DNase sensitive per nucleus regardless of differences in cell entry or nuclear accumulation at 24 h postinfection. The numbers above each bar represent the means of results from three independent experiments; error bars show standard errors.

We next asked whether the mutants had a defect in uncoating their DNA after nuclear entry. Infected nuclear extracts were prepared and either treated with Benzonase or left untreated, and the total amount of remaining DNA was quantitated by RT-PCR. When we compared the fractions of total nuclear viral DNAs that were DNase sensitive (that is, uncoated), we found that a significant fraction (0.5 to 0.8) of total nuclear DNA from all the mutant-infected nuclei was sensitive to Benzonase (Fig. 6A). One-way ANOVA showed no significant difference between the wt and mutants or the wt bound to A20 in their abilities to produce DNase-sensitive DNA in the nucleus.

We also calculated the number of uncoated (Benzonase-sensitive) genomes per nucleus (Fig. 6B). As expected, we found significantly lower numbers of uncoated genome copies of D528A and mut22 per nucleus. However, the absolute number of uncoated genome copies of all of the mutants should have been sufficient to produce a transduction frequency similar to that of the wt at an MOI of 10,000 particles per cell. Therefore, nuclear uncoating was not a viable explanation for the lack of gene expression in the mutants.

### Second-strand synthesis.

**FIG 7:**
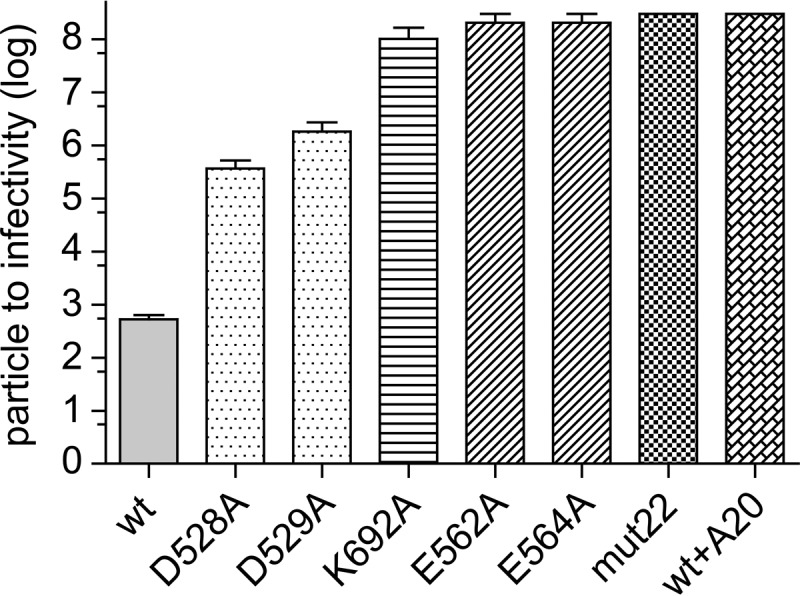
Particle-to-infectivity ratios of wt virus, mutant viruses, and wt virus bound to A20 carrying a self-complementary GFP cassette. The particle-to-infectivity ratio was determined as described in the legend of Fig. 2 except that a self-complementary GFP cassette was packaged in the wt and mutant capsids. Each bar represents the mean of data from 3 to 6 experiments; error bars indicate standard errors. Mutants with no error bars showed no detectable infectivity, and therefore, the particle-to-infectivity ratio is >3 × 10^8^, the limit of detection. One-way ANOVA was used to determine statistical significance (*F*[7,26] = 30; *P* < 0.0001; *n* = 3 to 6). Tukey's *post hoc* test results indicated that all comparisons with the wt had *P* values of <0.001 for all mutants and the wt bound to A20.

After uncoating, the next step in the AAV infection pathway is the synthesis of the second strand to form a duplex DNA molecule. Only then can transcription be initiated. To see if the mutants were defective for second-strand synthesis, we packaged a self-complementary DNA (scDNA) genome into each mutant capsid and measured the particle-to-infectivity ratio (Fig. 7). In principle, scDNA genomes would immediately form a duplex transcription template after uncoating and thus bypass the need for second-strand synthesis. We found that none of the mutants demonstrated improved transduction when scDNA genomes were packaged, and with one exception, K692A, the magnitude of the defect in infectivity with scDNA was similar to that seen with ssDNA genomes. Surprisingly, in the case of K692A, the defect in infectivity became ∼2 logs worse than that of capsids that carried a single-stranded genome. We concluded that second-strand synthesis was not likely to be the cause of the transduction phenotype in the mutants.

### Steady-state transcription at 24 h.

**FIG 8:**
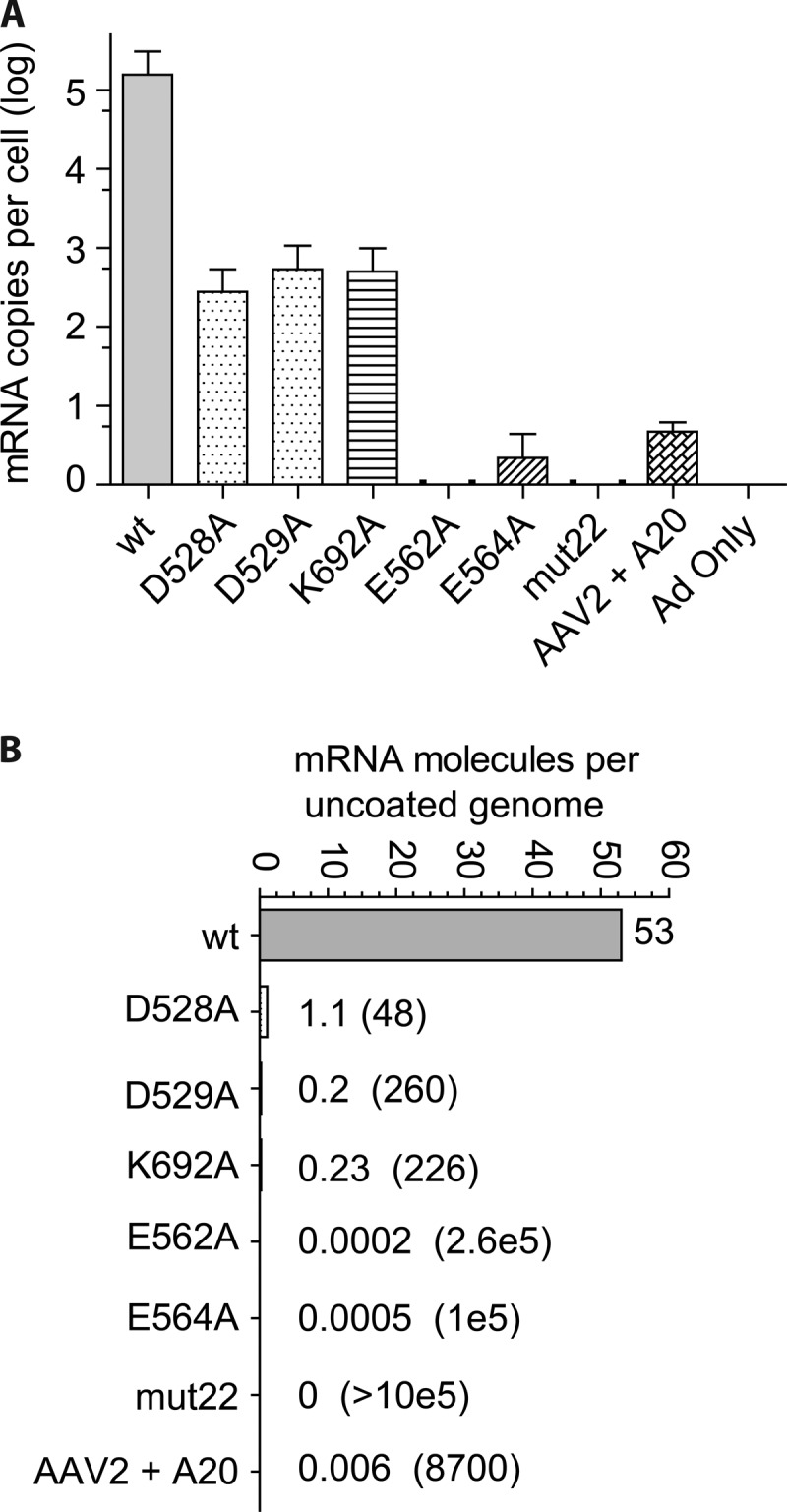
Steady-state levels of GFP mRNA produced by wt and mutant capsids. (A) mRNA was extracted from HeLa cells infected by wt and mutant capsids carrying single-stranded genomes at 24 h postinfection and measured by reverse transcriptase PCR. The mean number of copies of mRNA per cell is shown for each mutant, the wt, as well as the wt bound to the A20 antibody; error bars indicate standard errors (*n* = 3). (B) The mean number of copies of mRNA per cell, as shown in panel A, was normalized to the mean number of uncoated copies of DNA per nucleus (Fig. 6B) to determine the average number of mRNA copies synthesized per uncoated genome in the nucleus for the wt, the wt bound to A20, and the six mutants. For clarity, the number of mRNA copies synthesized per single copy of uncoated genome for each mutant is shown beside the mutant bars, and the fold reduction in transcription compared to the wt is shown in parentheses. No mRNA was detected for mut22.

Finally, we examined the accumulation of GFP transcripts at 24 h postinfection (Fig. 8). Mutants with the most severe defects in infectivity, E562A, E564A, and mut22, as well as the wt bound to A20 (Fig. 2) showed little to no mRNA accumulation (Fig. 8A). The defect in transcription was particularly striking for E564A, which had >50% more uncoated genomes per nucleus than the wt (Fig. 6B) but generated a 5-log-lower level of steady-state GFP mRNA per cell when normalized to the level of uncoated DNA per nucleus (Fig. 8B). In the case of mut22, no GFP mRNA was detected in spite of the fact that mut22 had levels of uncoated genomes per nucleus that were only 4.5-fold lower than those of the wt (compare Fig. 6B and 8). The same was true of the wt bound to A20, which had 3.6-fold fewer uncoated genomes per nucleus but generated 33,000-fold less mRNA per cell (Fig. 8A), which was 8,700-fold lower per uncoated genome than that of the wt (Fig. 8B). In addition, two mutants that had uncoated-genome levels comparable to those of the wt, D529A and K692A (Fig. 6B), generated ∼260- and 226-fold-lower levels of mRNA per uncoated genome, respectively (Fig. 8B). Finally, we note that D528A, which had an 11-fold defect in accumulating uncoated genomes in the nucleus (Fig. 8B), also had a 48-fold defect in mRNA synthesis per uncoated genome in the nucleus (with a range of 29- to 61-fold based on maximum and minimum values for uncoated DNA and mRNA for the wt and the D528A mutant). Thus, the primary defect in D528A, which also had a defect in cell entry (24 h) (Fig. 2) and a defect in nuclear entry (Fig. 5B), was a defect in mRNA transcription after nuclear entry. We concluded that the primary defect in all of the mutants that we examined, as well as in the wt virus bound to A20, was an inability to transcribe their genomes.

### Complementation of the mutant phenotype.

**FIG 9:**
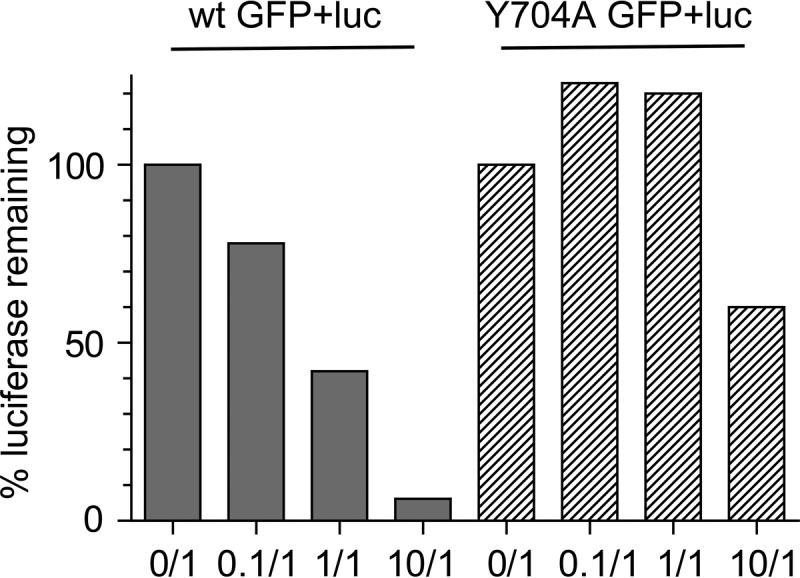
Inhibition of wt luciferase virus expression by wt GFP or Y704A GFP virus. HeLa cells were infected with the wt virus expressing the luciferase gene at an MOI of 1,000 and coinfected with either the wt GFP or Y704A GFP virus at a GFP-to-Luc ratio of 10:1, 1:1, 0.1:1, or 0:1. At 24 h postinfection, the expression of luciferase was measured as described in Materials and Methods.

To see if coinfection with a wt capsid could complement the Y704A dead-zone mutant, we infected cells with 1,000 particles per cell of the Y704A virus expressing GFP and coinfected these cells with 1,000 particles per cell of the wt virus expressing luciferase and mCherry. No GFP expression was seen from the Y704A packaged GFP genome (not shown). Thus, coinfection with the wt capsid did not complement the defect in Y704A. To see if Y704A infection would inhibit expression of a wt packaged genome, we performed the reciprocal experiment (Fig. 9). Cells were infected with wt Luc-Cherry virus and coinfected with either the wt GFP virus or the Y704A GFP virus at increasing MOIs. Coinfection with the wt GFP virus reduced luciferase expression in approximately a linear fashion. At a 1:1 ratio of wt Luc to wt GFP virus, luciferase expression was reduced ∼60%, suggesting that the two wt virus encapsidated genomes were competing for the available cell factors involved in cell entry, nuclear trafficking, and transcription. Similarly, at a 10:1 ratio, luciferase expression was reduced to 6% of the level seen when no competing wt GFP virus was added. In contrast, the Y704A GFP virus was unable to compete with luciferase expression at a 1:10 or 1:1 ratio, and reduced luciferase expression was seen only when the Y704A virus was added at an MOI of 10,000 particles per cell (10:1 ratio). We concluded that Y704A either could not effectively compete for transcriptional machinery or was present at a nuclear location that was different than that of the wt virus, where transcriptional machinery was inaccessible. It is also possible that the Y704A encapsidated genome was repressed by cellular enzymes, which did not affect wt packaged genomes.

## DISCUSSION

We have characterized 6 mutants within the 2-fold plateau of the AAV2 capsid for their ability to enter HeLa cells, traffic to the nucleus, uncoat their DNA, synthesize their complementary strand, and then transcribe their genome and express their gene. The mutants have particle-to-infectivity ratios that were 3 to >5 logs higher than those of wt capsids. We find that all of these mutants have a pronounced defect in their ability to promote the transcription of their genomes.

The most striking example of the transcription-negative phenotype was displayed by the D529A, K692A, D528A, and D564A mutants. These mutants entered the cell as well as the wt virus (Fig. 3), accumulated in the nucleus to approximately the same extent as the wt (Fig. 4 and 5), and accumulated approximately the same number of uncoated genomes per nucleus as the wt (Fig. 6B), and yet their genomes were transcribed a levels that were ∼300-fold lower (D529A and K692A) or 5 logs lower (D528A and D564A) than that of an identical genome delivered by a wt capsid (Fig. 8). The defect in transcription of these mutants correlated well with their decrease in transduction (compare Fig. 2 and 8). Moreover, when scDNA was packaged, there was no significant improvement in the particle-to-infectivity ratio (compare Fig. 2 and 7) compared to single-stranded packaged genomes, suggesting that second-strand synthesis was not the rate-limiting step for gene expression. Curiously, one of the mutants, K692A, was less infectious when self-cDNA was packaged.

The two remaining mutants, D528A and mut22, were found to also have a defect in cell entry (Fig. 3). However, even these two mutants, when normalized to the level of uncoated DNA per nucleus, accumulated 48-fold-lower (D529A) and undetectable (mut22) levels of GFP mRNA, respectively, compared to the wt (Fig. 8B). Thus, even these mutants had a severe defect in the transcription of their genomes once they were uncoated in the nucleus. Our results mimic those that we previously reported for the Y704A mutant (6), which is within 7.5 Å of D529 and E564. Taken together, our data suggest that this region of the capsid (Fig. 1), ∼30 Å in diameter and previously referred to as the dead zone (3), has a role in transcription of genomes packaged in AAV capsids.

**FIG 10:**
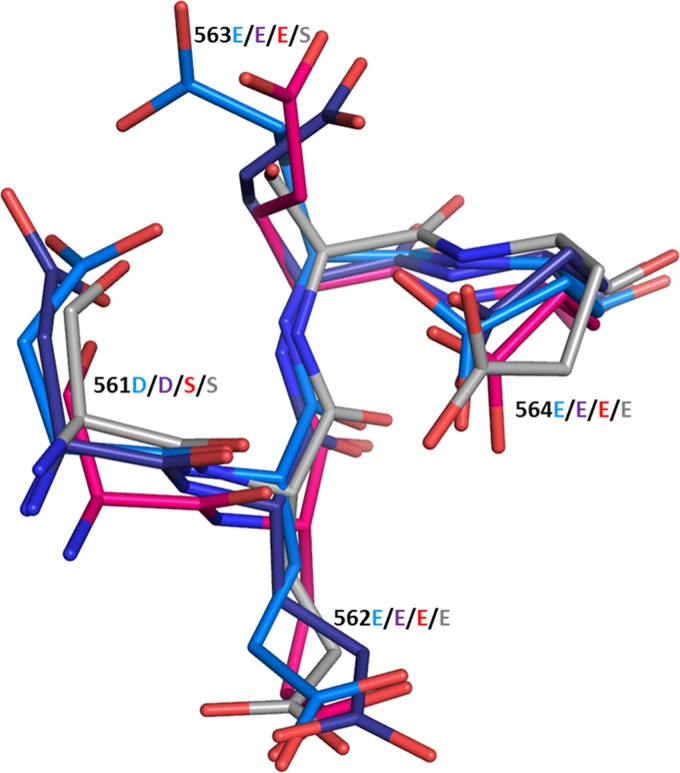
Structural comparison of the region composed of residues 562 and 564 of AAV1, -2, -4, and -5. The atomic alignments of AAV2 residues 562 and 564, which are universally conserved in all serotypes by amino acid alignment, were compared and found to have atomic structures that were virtually identical to those of both the closely related serotypes AAV1 (purple) and AAV2 (blue) and the distantly related serotypes AAV4 (red) and AAV5 (gray).

The seven amino acid positions that we have identified functionally map the transcription-negative region to a 30-Å region on the surface of the AAV capsid. All of these amino acids are highly conserved throughout >150 human, primate, murine, avian, and snake serotypes that have been sequenced. Table 1 summarizes the prevalence of each amino acid in the major primate clades when the capsid amino acid sequences are aligned by using FastA. Amino acid positions 562, 564, 692, and 704 are the most highly conserved positions. Positions 562, 564, and 692 are conserved in every serotype from every species, including avian and snake serotypes, that have been sequenced. Position 704 is missing only in primate clade D (AAV7), where Tyr is replaced with Phe, a structurally similar amino acid. The remaining amino acid positions are conserved in all clades except AAV4, AAV5, and avian and snake serotypes, which are the serotypes that are the most diverged from primate strains. The high level of conservation of these amino acid positions suggests that this capsid region performs an essential function for AAV infection. A structural comparison of the region composed of residues 562 and 564 of AAV2 with that of the related serotype AAV1 and the more diverged serotypes AAV4 and AAV5 revealed that the atomic structures of this region were virtually identical for all four serotypes (Fig. 10). A similar comparison of the region encompassing positions 528 and 529 (not shown) showed that the AAV1 and -2 atomic structures were virtually identical but that those of AAV4 and -5, whose amino acid sequences had diverged in this region, were dissimilar. Thus, the sequence alignment appeared to be a good indicator of structural similarity.

**TABLE 1:** Prevalence of each amino acid in the major primate clades

Clade (serotype[s])	Amino acid(s) at position(s):				
	269–271	528, 529	562, 564	692	704
A (AAV1, -6)	DNH	D, D	E, E	K	Y
B (AAV2)	DNH	D, D	E, E	K	Y
C (AAV3)	DNH	D, D	E, E	K	Y
D (AAV7)	DNT	D, D	E, E	K	F
E (AAV8, -10)	DNT	D, D	E, E	K	Y
F (AAV9)	DNT	E, G	E, E	K	Y
AAV4	SNT	P, A	E, E	K	Y
AAV5	ANA	L, Q	E, E	K	Y

In addition, we also examined the effect of neutralizing antibody A20 on infectivity. A20 is a conformational antibody that is often used to determine if AAV2 capsids are intact (9–11). A20 was previously shown to exert its effect on transduction in the nucleus (10), thereby showing that AAV enters the nucleus as an intact capsid. Previous work mapped the A20 binding site to a set of amino acids within the 2-fold plateau (3, 12), and mutational analysis implicated a set of 5 amino acids between K692 and mut22 on the capsid surface (Fig. 1, green residues). This suggested that gene expression might be inhibited by A20 by a mechanism related to that of the mutants. We confirmed that wt capsids bound to A20 enter the cell to the same extent as the wt (Fig. 3, black triangle) and accumulate ∼3-fold less uncoated DNA in the nucleus at 24 h than the wt (Fig. 6B). However, like the mutants in this region, A20, when bound to the capsid, inhibited transcription by almost 4 logs. This observation suggested two things. First, uncoating probably did not occur via disassembly of the 2-fold interface of the capsid; if it did, A20 could not still be bound and exert its effect on transcription in the nucleus. Second, the simplest explanation for the A20 effect was that one or more cellular factors could not gain access to the capsid at the 2-fold plateau, and this was likely the explanation for the mutant phenotypes as well.

Our data also demonstrated that the Y704A dead-zone mutant cannot be complemented by coinfection with the wt virus and that it does not exert a dominant negative effect on wt-mediated gene expression (Fig. 9). This suggests that either the mutant and wt capsids are located in different nuclear locations that contain different transcriptional control factors or the wt dead-zone region is *cis* active for the attraction of cellular transcription factors. The latter implies that AAV genomes remain associated with their capsids even after second-strand synthesis.

Several groups have identified nuclear factors that bind to the AAV capsid (13–17). Recently, Schreiber et al. (16) identified three proteins involved in the U2 snRNP complex as being inhibitors of AAV transduction. Treatment of cells with small interfering RNAs (siRNAs) for these proteins (PHF5A, SF3B1, and U2AF1) improved AAV transduction. Furthermore, two of the proteins, PHF5A and SF3B1, were coimmunoprecipitated with AAV capsids after infection. Knockdown of the U2 snRNP complex had no effect on viral entry, nuclear entry, uncoating, or second-strand synthesis. Rather, reductions of the levels of the U2 snRNP proteins significantly improved the transcription of the AAV genome after nuclear uncoating. A similar effect was seen with coinfection with adenovirus, which has been shown to reorganize the U2 snRNP complex (18). These two approaches, coinfection with adenovirus and knockdown of U2 snRNP proteins, are believed to work through the same mechanism (16). The effect of the U2 snRNP complex is reminiscent of what we saw with the mutants in the dead zone. Thus, a possible hypothesis might be that the virus capsid normally has a mechanism to bypass the inhibition of the snRNP complex, which is defective in the mutants. However, all of our experiments were done in the presence of adenovirus infection, and therefore, it is not clear if the transcription defect of the dead-zone mutants is related to the effect of U2 snRNPs on AAV transduction.

We also note that our previous work demonstrated that the dead-zone region of the capsid contained protease activity for external protein substrates. Mutation of residue E563, which is within 3 Å of Y704 and adjacent to E564, eliminates the capsid protease activity (19). Conceivably, the dead-zone mutants could also eliminate the protease activity, thereby preventing the digestion of a cellular factor that inhibits the transcription of AAV genomes.

Also, the dead zone, and specifically residues equivalent to Y704 and E563, has been shown in AAV8 (5) to undergo a structural change when the virus is exposed to acidic pHs that mimic the cellular endosomal environment. This structural change, or the absence of it, may also play a role in triggering transcription. It has been suggested, for example, that a pH-mediated structural change leads to the extrusion of the VP1 N-terminal amino acid sequences that contain the phospholipase activity, the nuclear localization signals, as well as other sequences that interact with cellular proteins (20, 21).

In conclusion, we have identified a new functional role for the 2-fold interface of the AAV capsid. Amino acid residues at this interface appear to have an essential role in promoting the transcription of AAV encapsidated genomes. Many groups have reported changes in viral propagation or gene expression that were due to mutations in the capsid structural proteins, and it has generally been assumed that these phenotypic changes were due to defects in cell entry, nuclear entry, or DNA uncoating. We have demonstrated, we believe for the first time, that mutations in capsid structural proteins can also have an effect on events after uncoating, specifically transcription, and we have independently confirmed this capsid function by demonstrating that an antibody that binds this region can reproduce the transcription-negative phenotype. Finally, the mutants reported here essentially map this functional region on the capsid surface. Our work suggests for the first time that capsid structural proteins are not simply delivery vehicles for nucleic acids but can also have a role in gene expression.
